# A Comparison of Inswing and Outswing Bowling Arm Kinematics of Pathway and Elite Male Fast Bowlers

**DOI:** 10.1002/ejsc.12321

**Published:** 2025-06-03

**Authors:** Cody Lindsay, Rian Crowther, Kane Middleton, Brad Clark, John Warmenhoven, Wayne Spratford

**Affiliations:** ^1^ Exercise and Sport Science and Clinical Exercise Physiology College of Nursing and Health Sciences Flinders University Adelaide Australia; ^2^ University of Canberra Research Institute for Sport and Exercise University of Canberra Canberra Australia; ^3^ Cricket Australia National Cricket Centre Albion Australia; ^4^ Sport, Student Services and Wellbeing Queensland University of Technology Brisbane Australia; ^5^ Sport, Performance, and Nutrition Research Group School of Allied Health, Human Services and Sport La Trobe University Melbourne Australia; ^6^ School of Sport, Exercise and Rehabilitation University of Technology Sydney Sydney Australia

**Keywords:** biomechanics, coaching, cricket, performance, swing bowling

## Abstract

Cricket fast bowlers can increase the difficulty for batters to accurately intercept their deliveries by swinging the ball. To generate swing, bowlers must use a technique that creates an angled and upright ball seam when it is released from the hand and projected towards the batter. The ability to create both inswing and outswing can provide bowlers with an advantage over opposition batters and requires a high level of skill, but little is known about the bowling actions to achieve this. This exploratory investigation aimed to compare the ball grip angle, bowling arm joint angles during the delivery stride and bowling arm segment orientations at the point of ball release of conventional new ball inswing and outswing deliveries. A three‐dimensional motion analysis system captured a group of 10 pathway and elite Australian fast bowlers delivering inswing and outswing. Statistical differences were observed in ball grip (*p* = 0.041), elbow pronation–supination (*p* = 0.050), shoulder adduction–abduction (*p* = 0.002), forearm lateral (*p* = 0.009) and hand lateral (*p* = 0.002) angles. These differences in bowling mechanics may assist bowlers in achieving the desired ball release positions and seam angles required to swing the ball in both directions. The findings of this study provide initial insight into the biomechanics of swing bowling, allowing for hypotheses that are generated from the data to be tested in future research and offering athletes and coaches strategies that can be employed to enhance performance.

**Summary**

- Fast bowlers employed similar bowling arm joint movements to swing the ball in both directions, but differences in ball grip angle and elbow supination were observed when comparing inswing and outswing deliveries.
- Different forearm and hand orientations at the point of ball release were observed when comparing swing directions.
- Combining technique and ball grip modifications are strategies that could be employed by bowlers to swing the ball both ways.

## 1 Introduction

Cricket is a contest between bat and ball with players categorised predominantly as either a batter or a bowler. Bowlers project the ball from one end of the wicket towards the batter positioned at the other end of the wicket, who aims to strike the ball with a bat to score runs. Therefore, bowlers are relied on to restrict run‐scoring and claim the wickets of opposition batters. Bowlers are categorised as either spin or fast bowlers. Spin bowlers impart side spin on the ball and use slow release speeds, whereas fast bowlers release the ball at higher speeds to limit the reaction time of opposition batters. A tactic that bowlers can employ with their deliveries to deceive batters, reduce run‐scoring and claim wickets is lateral deviation of the ball before it reaches the batter. This can be achieved before (swing and drift) and/or after (seam and spin) the ball bounces on the wicket, creating spatial uncertainty for batters who must predict the arrival location of the ball for an accurate interception (Müller and Abernethy, 2006). Fast bowlers can utilise swing, which is a laterally curved flight path of the ball before it bounces on the wicket. Fast bowlers can generate two directions of swing: inswing, which is lateral deviation towards the batter's body, and outswing, which is lateral deviation away from the batter's body.

In men's international cricket matches, researchers have highlighted that swinging deliveries resulted in reduced scoring and increased dismissal rates compared with other delivery types (S. Mehta et al. 2022). In a laboratory environment, Sarpeshkar et al. (2017) reported that swinging deliveries delayed the initiation of batters' movement patterns compared with straight deliveries. This delay reduced the quality of bat‐and‐ball contact, which has been reported as an indicator of batting skill (Houghton et al. 2011). Sarpeshkar et al. (2017) also reported that outswing deliveries caused a greater reduction in batting performance than inswing deliveries. This suggests that delivering outswing should be a priority for bowlers; however, generating swing in both directions can reduce predictability (Lindsay et al. 2022), exploit the weaknesses of different batters (Phillips et al. 2010) and provide an advantage over left‐ and right‐handed batters who use opposing body orientations in their batting stance (Connor et al. 2020). The ability to deliver both inswing and outswing requires a high level of skill, with subtle technique adjustments needed to release the ball in the desired position.

At the start of each innings, the fielding team receives a new cricket ball with a pronounced seam and smooth leather surface that is conducive to swing bowling. When the ball is in this condition, fast bowlers can manipulate the seam orientation to cause swing towards the direction of the seam angle, referred to as conventional swing bowling (R. D. Mehta 2005). To create conventional swing, bowlers must release the ball with an upright and angled seam position. Outswing requires a seam orientation that is angled towards the slip fielding position located behind the batter on the offside of the wicket (e.g., approximately 20° counterclockwise in the transverse plane for a right‐handed batter when viewed from the bowler's end of the wicket) (R. D. Mehta 2005). Conversely, inswing requires a seam orientation that is angled towards the leg slip fielding position located behind the batter on the onside of the wicket (e.g., approximately 20° clockwise in the transverse plane for a right‐handed batter when viewed from the bowler's end of the wicket) (R. D. Mehta 2005). To create these seam orientations, the ball grip and technique employed by fast bowlers are crucial (Lindsay et al. 2022; Lindsay and Spratford 2020, 2021). The majority of fast bowling biomechanics research has focused on technique variables related to injury (Farhart et al. 2023) and ball release speed (Ramachandran et al. 2024), but swing bowling has received less attention.

To our knowledge, only two studies have quantitatively investigated the technique kinematics of swing bowling in elite cricket (Lindsay et al. 2023; Lindsay, Crowther, Clark, et al. 2024). One study investigated pelvis and upper‐body kinematics, reporting relative stability in the larger proximal body segments of the pelvis and torso compared with the more distal body segments of the bowling arm, which showed higher amounts of variability (Lindsay, Crowther, Clark, et al. 2024). These are important considerations for coaches when designing training interventions, but the authors presented the kinematics at the discrete time point of ball release. This approach discarded a large amount of data that would be valuable in understanding the movement patterns of bowlers used to produce an angled seam orientation for inswing and outswing deliveries. An earlier study using time series data during the delivery stride showed consistency with these outcomes of high movement variability at the elbow and wrist for outswing deliveries but unfortunately did not measure inswing techniques (Lindsay et al. 2023). The technical attributes associated with swinging the ball in both directions remain unclear.

Therefore, this study aimed to compare ball grip angles, bowling arm joint angles during the delivery stride and bowling arm segment orientations at the point of ball release between inswing and outswing deliveries in a group of pathway and elite Australian fast bowlers. Given that the research investigating swing bowling technique is already sparse and to date there are limited studies demonstrating continuum‐based (e.g., functional data analysis, statistical parametric mapping, etc.) methods in the context of swing bowling, this current manuscript will focus on an exploratory research study and will frame directional hypotheses to be tested at the conclusion of the paper (Pataky, Vanrenterghem, Robinson 2016). Developing an understanding of the bowling arm movements employed by bowlers to deliver inswing and outswing will provide direction for future studies, allow for hypotheses that are generated from data to be tested and guide coaches to implement training strategies to improve the ability of bowlers to swing the ball both ways.

## 2 Materials and Methods

### 2.1 Participants

Twelve Australian fast bowlers participated in this study. The criteria for inclusion within the comparative analysis between inswing and outswing deliveries were 0.2° of swing in both directions. After data processing, two bowlers were removed from the group‐level analyses for failing to deliver inswing. The data of 10 Australian male elite (first‐class cricket level and above: senior state, *n* = 5) and pathway (up to first‐class cricket level: state U19, *n* = 1, international U19, *n* = 4) fast bowlers (age 20.1 ± 1.6 years, mass 86.4 ± 10.1 kg, height 193.1 ± 5.2 cm) were included in the comparative group analyses. All bowlers used legal bowling actions as defined by the International Cricket Council (≤ 15° elbow extension occurring between upper‐arm horizontal and ball release). Ethics approval was granted by the University of Canberra Human Research Ethics Committee (approval #4645). All participants provided informed consent.

### 2.2 Data Collection

Data collection occurred at an indoor training facility containing artificial wickets and space for full‐length run‐ups. Retroreflective markers (14‐mm diameter) were affixed to participants according to the University of Western Australia (UWA) full‐body marker set (Besier et al. 2003; Campbell, Lloyd, et al. 2009; Campbell, Alderson et al. 2009; Chin et al. 2010; Lloyd et al. 2000; Wells et al. 2018). Marker placements were conducted by one researcher for all participants. Three 0.1‐mm thick, 13‐mm diameter and 0.02‐g retroreflective tape patches (3M Scotchlite High Grain Reflective Sheeting 7610) were affixed to new red Kookaburra Turf cricket balls (mass 0.156 kg) in locations used in previous research (Lindsay and Spratford 2020; Spratford et al. 2018). This tape allowed the ball to be tracked during flight while minimising aerodynamic interference compared with three‐dimensional markers commonly used in ball‐tracking research (Spratford et al. 2018). After marker placement, participants warmed up using a self‐selected protocol and completed static and functional movement trials to calculate joint centres and axes of rotation.

**FIGURE 1:**
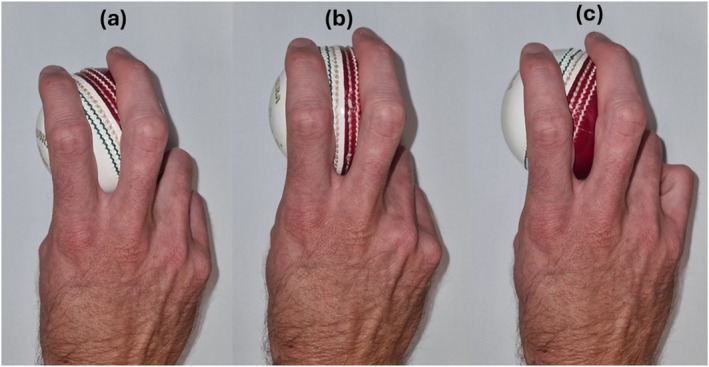
Examples of (a) a negative angle cross‐seam grip, (b) a zero‐degree straight‐seam grip and (c) a positive angle cross‐seam grip with the anterior portion of the seam angled towards the left, parallel and to the right of the index and middle fingers, respectively, as viewed from the bowler’s end of the wicket.

Each participant was right‐handed and completed 18 deliveries/trials with one new cricket ball. Participants gripped the ball so the side with two markers was facing the intended swing direction but were otherwise free to use a grip of their choice. This instruction was provided to minimise the aerodynamic interference on the ball during flight, as the presence of an angled seam on the side with two markers disturbed airflow before reaching the attached markers. The presence of one marker on the opposite side of the ball allowed air to travel around a section of the ball before contacting the marker, maintaining airflow asymmetry and swing (R. D. Mehta 2005; Lindsay, Crowther, Clark, et al. 2024). The ball grip angle was measured using a protractor before each delivery. Zero degrees represented a seam parallel to the fingers (straight seam grip), with positive and negative angles representing a seam angled towards the right and left (cross‐seam grip), respectively, when viewed from the bowler's end of the wicket (Figure 1). Participants were instructed to replicate match intensity and pitch the ball on a good‐to‐full length (three to seven metres from the batter's stumps (Justham et al. 2010)) as though bowling to a right‐handed batter. The testing protocol consisted of nine outswing and nine inswing deliveries. The delivery order was block randomised, and the swing direction was counterbalanced. Participants were instructed to deliver, in order, either six inswing, six outswing, three inswing and three outswing deliveries or the same delivery structure with the opposite swing direction. After each delivery, the ball was assessed for damaged markers and scuffs. If required, the markers were replaced and the ball was polished to maintain a smooth surface. Body and ball marker trajectories were captured using a 40‐camera (MX T40) Vicon motion analysis system (Oxford Metrics Ltd., Oxford, UK) sampling at 250 Hz. The capture volume was large enough to capture the pre‐delivery stride, delivery stride and entire ball flight when pitched at least two metres from the batter's stumps.

### 2.3 Data Processing

Body and ball marker trajectories were reconstructed and labelled using Vicon Nexus software (v2.12, Oxford Metrics Ltd., Oxford, UK). Body marker trajectories were filtered using a fourth‐order zero‐lag Butterworth filter with a cut‐off frequency of 14 Hz, after a residual analysis and visual inspection of the data (Winter 2009). Body kinematics were calculated using the valid and reliable UWA upper‐ and lower‐body models (Besier et al. 2003; Campbell, Lloyd et al. 2009; Campbell, Alderson et al. 2009; Chin et al. 2010; Lloyd et al. 2000; Wells et al. 2018). Kinematics were extracted and normalised to 100% (101 points) of the delivery stride between back foot contact and ball release using a custom cubic spline Python script (v3.11, Python Software Foundation, Wilmington, DE, USA). Back foot contact was defined as the frame when the right foot marker trajectories were visually observed to change due to foot contact with the ground after the pre‐delivery stride (Worthington et al. 2013). Ball release was defined as the frame when the distance between a ball marker and a marker placed on the head of the third metacarpal of the bowling hand increased by greater than 20 mm relative to the previous frame (Worthington et al. 2013).

**FIGURE 2:**
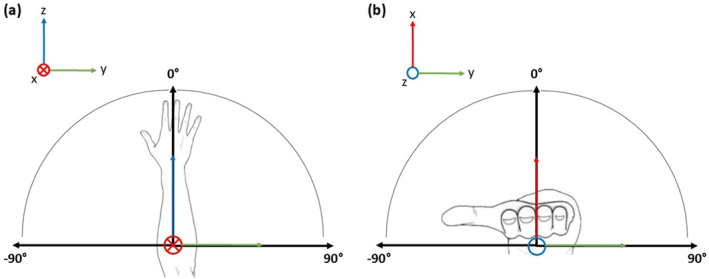
Forearm and hand segment angles relative to the global axes at the point of ball release. (a) Lateral forearm and hand angle relative to the anteroposterior axis when viewed posteriorly to the bowler and (b) hand rotation angle relative to the global longitudinal axis when viewed superior to the bowler. The coloured portions of the figure indicate the global coordinate axes: red for the *x*‐axis (anteroposterior), green for the *y*‐axis (mediolateral) and blue for the *z*‐axis (longitudinal). Arrows show the positive direction of each axis. A closed circle with a cross indicates a positive axis directed into the page, whereas an open circle indicates a positive axis directed out of the page.

Bowling arm joint and segment angles were calculated. Joint angles were reported using the International Society of Biomechanics recommendations (Wu et al. 2005). Positive angles represented shoulder flexion, shoulder adduction, elbow flexion, elbow pronation, wrist flexion and wrist adduction. Negative angles represented shoulder extension, shoulder abduction, elbow extension, elbow supination, wrist extension and wrist abduction. Segment angles were measured relative to the global axes. The lateral angle measured the segment orientation in the anteroposterior axis. Zero degrees was orientated perpendicular to the ground, increasing positively in a clockwise direction and negatively in a counterclockwise direction when viewed from behind the bowler (Figure 2a). The rotation angle measured segment orientation in the longitudinal axis. Zero degrees was orientated down the wicket towards the batter's end, increasing positively in a clockwise direction and negatively in a counterclockwise direction when viewed from above the wicket (Figure 2b). Joint angles are presented as time‐normalised data of the delivery stride, and segment angles are presented at the point of ball release. These variables offer insight into movement patterns of the bowling arm during the delivery stride and the bowling arm orientation at the point of ball release. These factors were suggested to be important technique parameters for swing bowling in previous research (Lindsay, Crowther, Middleton, et al. 2024).

Delivery swing was calculated using the *x*, *y* coordinates of the ball. A vector was created using the coordinates at release and one frame post‐release. A second vector was created using the coordinates of the ball at release and the pitch location on the wicket. The angle between the two vectors was calculated as a measure of swing for each delivery (Lindsay and Spratford 2020). To be included in the analyses, deliveries must have swung at least 0.2°. This threshold was chosen because it represents the minimum magnitude of swing likely to result in a performance reduction in batters. When pitched on a good length, six metres from the batter's stumps, this amount of swing results in a lateral deviation of approximately three centimetres and can result in miss‐hits or missed shots of batters (Lindsay, Crowther, Clark, et al. 2024; Justham et al. 2010; Peploe et al. 2018). Outswing and inswing deliveries swung to the left and right, respectively, relative to the original direction of travel when viewed from the bowler's end of the wicket and for a right‐handed batter.

### 2.4 Data Analysis

Python (v3.11, Python Software Foundation, Wilmington, DE, USA) was used for all analyses. Participant means were calculated and used to calculate group descriptive values (means and standard deviations) of discrete data for ball grip, segment orientations and delivery swing for inswing and outswing deliveries. Two‐tailed paired‐sample *t*‐tests statistically compared ball grips and segment angles between swing directions. Cohen's effect sizes (*d*) were calculated to functionally compare discrete data with classifications of trivial (*d* ≤ 0.2), small (*d* = 0.2–0.49), medium (*d* = 0.5–0.79) and large (*d* ≥ 0.8) used (Cohen 1992). Differences between swing directions across the time‐series data were examined using one‐dimensional statistical parametric mapping (SPM) two‐tailed paired‐sample *t*‐tests using open‐source code (www.spm1d.org) (Pataky 2010). Significance was set at *p* ≤ 0.05 for all statistical analyses.

## 3 Results

Group means, standard deviations, *p*‐values and effect sizes of discrete data comparing inswing and outswing deliveries are presented in Table 1. Significant differences were found in ball grip (*p* = 0.041), hand lateral angle (*p* = 0.002) and forearm lateral angle (*p* = 0.009). On average, bowlers produced 0.58° and 0.80° of swing for inswing and outswing deliveries, respectively.

**TABLE 1:** Group means ± standard deviations for ball grip angle, delivery swing and segment orientation at the point of ball release for inswing and outswing deliveries.

Variable	Inswing	Outswing	*p*‐value	*d*
Ball grip (°)	8.07 ± 9.51	−5.72 ± 13.77	0.041^a^	1.166^c^
Hand rotation angle (°)	18.85 ± 11.39	15.52 ± 13.40	0.070	0.268
Hand lateral angle (°)	−6.48 ± 8.71	−1.23 ± 10.16	0.002^a^	0.555^b^
Forearm lateral angle (°)	−6.87 ± 8.01	−4.42 ± 7.52	0.009^a^	0.315
Swing (°)	0.58 ± 0.29	0.80 ± 0.29	—	—

^a^ Significant *p* ≤ 0.05.

^b^ Medium *d* = 0.50–0.79.

^c^ Large *d* ≥ 0.80.

Individual participant (*n* = 2) means for ball grip angle and segment orientations for unsuccessful inswing deliveries are presented in Table 2. Compared to successful group inswing deliveries, bowlers exhibited similar ball grip and forearm lateral angles, whereas larger differences were observed in hand rotation and lateral angles.

**TABLE 2:** Means for ball grip angle and segment orientation at the point of ball release for unsuccessful inswing deliveries.

Variable	Participant 1	Participant 2
Ball grip (°)	12.44	10.75
Hand rotation angle (°)	−5.96	−3.21
Hand lateral angle (°)	9.76	12.63
Forearm lateral angle (°)	−14.50	−0.49

*Note:* Values are presented for individual participants.

**FIGURE 3:**
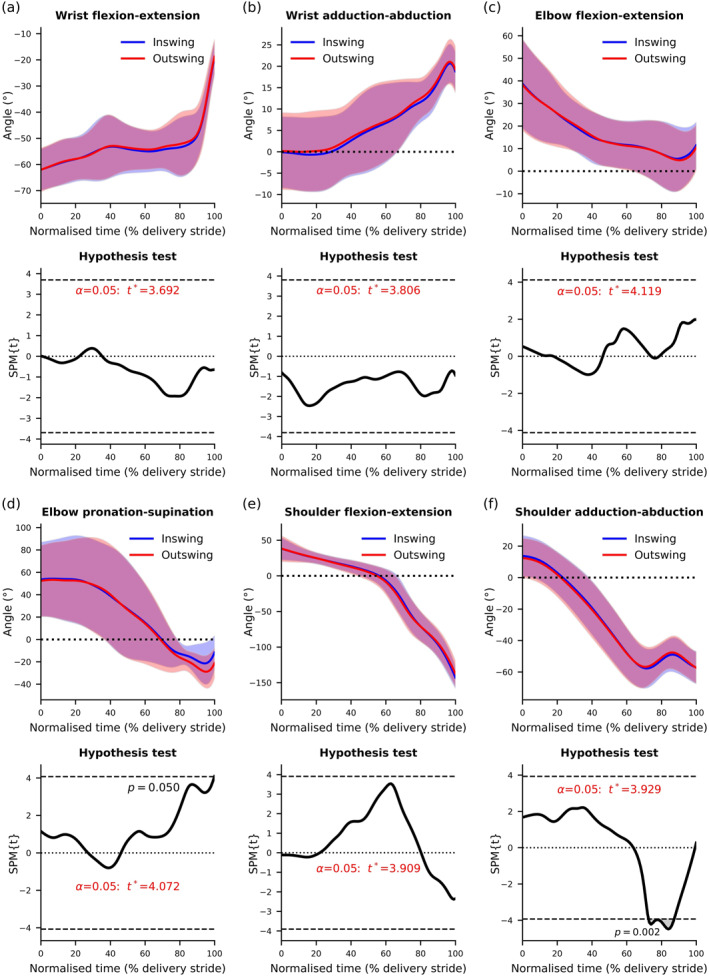
Mean data and SPM results for wrist, elbow and shoulder angles. The upper section of each subfigure presents mean data ± one standard deviation throughout the delivery stride, represented by the solid lines and shaded areas, respectively. The lower portion of each subfigure displays the SPM results where the critical *t*‐value threshold is presented in red text and by the dashed lines above and below zero. Where the *t*‐value exceeded the threshold, significance was reached and is highlighted by grey‐shaded areas.

Group means, standard deviations and SPM two‐tailed paired‐sample *t*‐test results for wrist, elbow and shoulder angle time‐series data of inswing and outswing deliveries are presented in Figure 3. Compared with inswing, outswing deliveries had significantly greater elbow supination (*p* = 0.050) at the point of ball release and significantly less shoulder abduction (*p* = 0.020) between approximately 75% and 85% of the delivery stride.

## 4 Discussion

This exploratory study compared ball grip angles, bowling arm segment orientations at the point of ball release and time series data of bowling arm joint angles during the delivery stride of inswing and outswing bowling. The results indicate that differences in ball grip, elbow pronation–supination and shoulder adduction–abduction were employed by bowlers to create both directions of swing. The results of the discrete data indicate different forearm and hand orientations at the point of ball release when comparing inswing and outswing.

The bowlers included in the comparative analyses successfully created both directions of swing. On average, bowlers generated 0.58° and 0.80° of swing for inswing and outswing (Table 1), resulting in a lateral deviation of approximately 10 and 13 cm, respectively, when pitched on a good length, six metres from the batter's stumps (Lindsay and Spratford 2020; Justham et al. 2010). Combining their technique and ball grip allowed bowlers to create an angled and upright seam orientation that is required to generate swing (Lindsay et al. 2022; Jinji et al. 2011). Most bowlers gripped the ball with a shallow seam angle in the direction of the intended swing, but variation was observed in the results (inswing: 8.07 ± 9.51°, outswing: −5.72 ± 13.77°, Table 1). This variance aligns with coaches' perceptions that the ball grip should be individualised to suit each athlete's technique (Lindsay, Crowther, Middleton, et al. 2024). Bowlers with intrinsic dynamics that lead to movement patterns allowing them to more easily deliver one direction of swing may find it difficult to swing the ball both ways without making large changes to their ball grip (Lindsay, Crowther, Middleton, et al. 2024; Glazier and Mehdizadeh 2019), explaining the variation in this measure. However, in the performance environment, there need to be tactical considerations for altering the seam angle, as this information is typically available for the batter to observe earlier in the bowler's run‐up phase (Müller et al. 2006). Therefore, employing variations in movement patterns may create opportunities for bowlers to generate an angled seam orientation to swing the ball both ways while also minimising the anticipatory information available to the batter before release.

The time series joint angles indicate that bowlers used similar movement patterns to swing the ball both ways (Figure 3). During approximately 75%–85% of the delivery stride, bowlers demonstrated statistically different shoulder adduction–abduction angles (*p* = 0.002) when comparing swing directions. However, this returned to a similar value at ball release (Figure 3f). Shoulder abduction is a strategy that can be employed to alter bowling arm orientation, and a statistical difference (*p* = 0.009) was observed in the forearm lateral angle (see Figure 2 for angle conventions) at the point of ball release. In this study, the forearm lateral angle remained inside perpendicular to the ground for both swing directions in most participants (inswing *n* = 7, outswing *n* = 7), but outswing deliveries displayed a slightly more outward orientation (inswing = −6.87° ± 8.01°, outswing = −4.42° ± 7.52°, Table 1). Bowlers and coaches have previously suggested that a bowling arm orientation inside perpendicular is suited to inswing and a bowling arm outside perpendicular is suited to outswing (Lindsay, Crowther, Middleton, et al. 2024). Although the bowlers in this study maintained an arm orientation inside perpendicular for both swing directions, the statistical difference does indicate some support for these claims. Technical parameters, other than shoulder abduction, that can influence the global forearm orientation include thorax lateral flexion (Escamilla et al. 2023; Fleisig et al. 2009) and elbow flexion due to the externally rotated position of the shoulder at ball release during fast bowling (Persad 2016). Despite bowlers demonstrating greater elbow flexion at the point of ball release for inswing deliveries, the difference was not statistically significant (Figure 3c), and thorax lateral flexion was not investigated in this study. These are strategies that can move the forearm further inside a perpendicular orientation for inswing deliveries but require further investigation.

The small difference (*d* = 0.315, mean difference = 2.45°) in forearm lateral angles highlights the relative stability of this technique parameter across swing directions for the participants included in the comparative analyses (Lindsay, Crowther, Clark, et al. 2024). The perceptions of athletes do not always match reality (Crowther et al. 2021), and it has been shown that a gap exists between perceived and actual movement (Giblin et al. 2014). The arm orientation suggested by bowlers and coaches in previous research (Lindsay, Crowther, Middleton, et al. 2024) could be used as a cue by athletes to produce a small technique change that, when combined with changes to other joints and segments, may create the desired seam orientation for inswing and outswing. This may not create a large difference in bowling arm kinematics for bowlers who predominantly swing the ball in one direction, but it may be enough to produce a change in seam angle to swing the ball in their non‐favoured direction. The influence of cues on bowling kinematics should be considered in future research.

The bowlers employed different movements of elbow pronation–supination to produce both directions of swing. Changes in mean pronation–supination angle can be observed from approximately 70% of the delivery stride, with outswing deliveries having statistically greater supination at ball release (*p* = 0.05, Figure 3d). Rotation at the elbow changes hand orientation, and the movement used by the participants in this study offers support to the suggestion in previous research that some bowlers attempt to wrap their hand ‘around the ball’ at release (Lindsay, Crowther, Middleton, et al. 2024). Elite fast bowlers discussed this as a strategy to assist them in positioning their hand and fingers on the inside and outside portions of the ball for inswing and outswing, respectively (Lindsay, Crowther, Middleton, et al. 2024). Furthermore, some bowlers discussed that they aim for the index and middle fingers to be the final point of contact with the ball for inswing and outswing, respectively (Lindsay, Crowther, Middleton, et al. 2024), highlighting the perceived importance of finger position for swing bowling. Statistical differences in the hand rotation angle between inswing and outswing were not observed in this study (*p* = 0.070, *d* = 0.268, inswing: 18.85 ± 11.39°, outswing: 15.52 ± 13.40°, Table 1). However, this measure displayed variation, demonstrated by large standard deviations, and bowlers likely employed individualised positions with subtle differences between swing directions (Lindsay, Crowther, Clark, et al. 2024), whereas others may use it as a cue like the suggestion for arm orientation. The hand orientation required to create an angled seam at ball release will depend on the individualised grip used by bowlers (Lindsay, Crowther, Middleton, et al. 2024). Elbow pronation–supination appears to be a strategy that bowlers can employ to swing the ball both ways.

The position of the fingers relative to the ball seam may be an important factor in creating swing. Although the hand rotation angle was not statistically different (*p* = 0.070) between swing directions in this study (Table 1), bowlers may have manoeuvred their fingers, the end effectors, to the desired position to create an upright and angled seam orientation (Lindsay, Crowther, Middleton, et al. 2024). This suggestion is supported by research reporting that finger kinematics influence ball spin in baseball pitch variations (Matsuo et al. 2018; Wang et al. 2013), similar to the differences in seam orientation required for inswing and outswing. Flexion, extension, adduction and abduction of the metacarpophalangeal joints occur independently of wrist movements (Wang et al. 2013) and may be employed by fast bowlers to manoeuvre the index and middle fingers to the desired location relative to the ball seam. Finger kinematics were not investigated in this study and should be considered in future research.

Two bowlers who failed to deliver inswing were not included in the comparative analyses (Table 2). Compared to the discrete inswing group kinematics, the unsuccessful bowlers used a similar ball grip (unsuccessful bowlers: 12.44° and 10.75°, group mean: 8.07° ± 9.51°) and forearm lateral angle (unsuccessful bowlers: −14.50° and −0.49°, group mean: −6.87° ± 8.01°), within one standard deviation of group results. However, differences greater than one standard deviation of group data were observed in the hand rotation angle (unsuccessful bowlers: −5.96° and −3.21°, group mean: 18.85° ± 11.39°) and hand lateral angle (unsuccessful bowlers: 9.76° and 12.63°, group mean: −6.48° ± 8.71°). The unsuccessful bowlers released the ball with the palm orientated towards the offside of the wicket, in the global longitudinal axis (see Figure 3b), and tilted laterally towards the onside of the wicket, in the global anteroposterior axis (see Figure 3a). Similar hand orientations have been reported for lower‐level bowlers delivering outswing (Lindsay and Spratford 2020), and we believe that the failure to deliver inswing in these participants was due to their hand orientation at ball release. This suggestion can be explained by baseball researchers reporting that the spin axis imparted on thrown balls is parallel to the plane created by the palm and fingers (Jinji et al. 2011). Inswing deliveries require a seam orientation and spin axis opposite to the hand position used by these bowlers. Therefore, it is unlikely that they could manoeuvre their fingers and the seam to be rotated towards the onside at release to deliver inswing. Although further research is needed to understand the role of the hand and fingers in swing bowling, this data offers further support to our suggestion that finger position is crucial for swing bowling.

This study has applications that could benefit coaches and athletes. This study identified that bowlers could draw on different elbow pronation–supination movements to swing the ball in opposite directions. Coaches and bowlers could explore this movement variation as a strategy to manoeuvre the position of the hand, fingers and ball seam. This study also identified differences in forearm and hand orientations between inswing and outswing. Although these differences were small and remained inside perpendicular for both swing directions, outswing deliveries displayed a more outward orientation, offering some support to the beliefs of elite bowlers and coaches (Lindsay, Crowther, Middleton, et al. 2024). Bowlers may benefit from cues relating to arm and hand position to encourage small technique adjustments that are enough to alter the seam angle, allowing the ball to swing both ways. Although the bowlers in this study demonstrated differences in these technical parameters when swinging the ball both ways, bowlers have been shown to use individualised techniques (Lindsay, Crowther, Middleton, et al. 2024; Lindsay, Crowther, Clark, et al. 2024). This aligns with the importance of not applying a ‘one‐size‐fits‐all’ approach to improve performance (Lindsay, Crowther, Middleton, et al. 2024; Glazier and Mehdizadeh 2019). The findings of this study serve as a starting point for athletes and coaches, but individual technique parameters must be considered when applying this information in practice (Lindsay, Crowther, Middleton, et al. 2024; Lindsay, Crowther, Clark, et al. 2024).

This study has limitations that must be considered. This study used a group‐level analysis, and conducting individual analyses would provide a clearer understanding of individual technique attributes and movement variability that allow bowlers to swing the ball both ways (Lindsay, Crowther, Middleton, et al. 2024; Glazier and Mehdizadeh 2019). This study did not investigate movement velocity during the delivery stride. Joint and segment angular velocities and the movement phase time (i.e., time between backfoot contact and ball release) would provide greater insight into the rate of movement used by bowlers to achieve their release position and should be included in future studies. This study did not investigate finger kinematics, and this information is needed to understand how the ball is released from the hand when creating swing. Researchers have previously used motion analysis to capture finger kinematics of baseball pitching (Wang et al. 2013), and future fast bowling studies could benefit from this inclusion. Further, data collection occurred without a batter present, reducing the replication of match conditions. In match environments, fast bowlers may employ technique variations not reported in this study to deceive opposition batters who attempt to anticipate delivery types and swing directions (Marshall et al. 2024).

Finally, given that this was an exploratory study, it is important to treat the findings with some level of caution, particularly until outcomes are replicated with a larger and adequately powered sample (using the directional hypotheses framed in the conclusion). Additionally, some careful consideration of powering 1D studies should be considered (e.g., if SPM is identified as a suitable framework for analysis as a part of future studies, approaches to calculating power for 1D experiments could be considered (Pataky 2017; Pataky, Robinson, Vanrenterghem 2016)). Further to this, given the sample of 10 bowlers, simpler statistical models (i.e., 1D SPM paired‐sample *t*‐tests) were used. In a future study aiming to replicate these outcomes, other more appropriate multivariate methods (i.e., function on scalar regression models (Ramsay and Silverman 2005)) could be used. These would, however, require a much larger sample to adequately assess the multivariate effects of multiple joints and segments on swing.

## 5 Conclusion

This study compared the ball grip angle, forearm and hand orientation at the point of ball release and bowling arm joint angles during the delivery stride of conventional new ball inswing and outswing bowling. On average, bowlers used shallow cross‐seam grips with the primary seam of the ball angled towards the direction of swing. SPM results revealed significant differences in shoulder adduction–abduction angle during approximately 75%–85% of the delivery stride and in elbow pronation–supination angle at ball release. These were accompanied by differences in forearm and hand orientation at the point of ball release when comparing inswing and outswing deliveries. These kinematic differences could allow bowlers to generate the seam orientation required to swing the ball in both directions. This research could guide training strategies and future studies to improve and understand swing bowling performance.

As such, the following directional hypotheses are provided for future research studies to test in independent studies:

1. For discrete data, the ball grip angle will be statistically larger, and the forearm lateral angle and the hand lateral angle will be statistically lower for inswing deliveries.
2. For 1D analyses, outswing deliveries will possess statistically greater elbow supination around the point of ball release and less shoulder abduction late in the delivery stride.

## Ethics Statement

This research was approved by the University of Canberra Human Research Ethics Committee (approval #4645).

## Conflicts of Interest

The authors declare no conflicts of interest.
